# Comparative Cost Analysis of Four Different Computer-Assisted Technologies to Implant a Total Knee Arthroplasty over Conventional Instrumentation

**DOI:** 10.3390/jpm12020184

**Published:** 2022-01-30

**Authors:** Bernhard Christen, Lars Tanner, Max Ettinger, Michel P. Bonnin, Peter P. Koch, Tilman Calliess

**Affiliations:** 1 Articon Spezialpraxis für Gelenkchirurgie, Salem-Spital, 3013 Berne, Switzerland; b.christen@articon.ch; 2 Department of Automotive Engineering, Bern University of Applied Science, Campus Biel, 2502 Biel, Switzerland; lars.tanner@students.bfh.ch; 3 Department for Orthopaedic Surgery, Hannover Medical School at Annastift Hospital, 30625 Hanover, Germany; max@ettinger.info; 4 Centre Orthopedique Santy, 69008 Lyon, France; bonnin.michel@gmail.com; 5 Clinic for Orthopedics and Traumatology, Cantonal Hospital Winterthur, 8400 Winterthur, Switzerland; peter.koch@ksw.ch

**Keywords:** robotics, navigation, patient-specific instruments, cost analysis

## Abstract

Several computer-assisted technologies, such as navigation and robotics, have been introduced to Total Knee Arthroplasty (TKA) in order to increase surgical precision and reduce complications. However, these technologies are often criticized due to the increased costs and effort associated with them; however, comparative data are missing. The aim of the present study was to evaluate differences in intraoperative workflows and the related perioperative cost-profiles of four current computer-assisted technologies, used to implant a TKA, in order to gain a comparison to conventional instrumentation. For the cost analysis, additional preoperative imaging and instruments, increased operating room (OR) and planning-time, and expenditures for technical support of the equipment and disposals were calculated, in comparison to conventional TKA, for (1) standard computer-navigation, (2) patient specific instruments (PSI), (3) image-based robotic assistance, and (4) imageless robotic assistance. Workflows at four expert centers which use these technologies were reviewed by an independent observer. The total cost calculation was based on a 125 TKA per year unit in Switzerland. Computer-navigation resulted in 14 min (+23%) increased surgery time and, overall, USD 650 in additional costs. PSI technology saved 5 min (8%) OR time but it created USD 1520 in expenditures for imaging and disposals. The image-based robotic system was the most expensive technology; it created overall additional costs of USD 2600, which predominately resulted from technical support, disposals, the CT-Scan, and 14 min of increased OR time. The imageless robotic assistance resulted in the largest increase in OR-time, as it resulted in an additional 25 min (+42%) on average. Overall, additional costs of USD 1530 were calculated. Every one of the assistive technologies in this study increased the total cost of TKA when compared to a conventional technique, and the most important variables, related to cost, were technical support and additional disposables. The longer surgical times and additional surgical trays required for the techniques had a marginal effect on overall costs. This comparative cost analysis gives valuable information for future efforts to calculate the real costs of these technologies and the subsequent return on investment of each technique.

## 1. Introduction

The conventional instrumentation used to position a total knee arthroplasty (TKA) in a reproducible manor was established in the 1980s. It currently represents the standard method in the majority of implantations performed globally. However, in the years following its establishment, several computer-assisted technologies have been introduced as alternative or supplementary methods. The major objective of these technologies is to increase surgical precision while reducing the outliers and complications associated with conventional instrumentation. Firstly, passive computer-navigation tools (CAS) used to position the cutting jigs with reference to a computed knee model were introduced in the operating room (OR) in the late 1990is. Currently, there is a breadth of evidence available in the literature suggesting that, indeed, the surgical precision and implant alignment can be improved [1,2,3]. In radiological outcome analysis, a significant improvement in the component position in the coronal and sagittal plane is described, compared to conventional instrumentation, and it reduced outliers in the overall limb alignment for the accepted boundaries of ±3° to the mechanical axis. However, this effect could not be shown in the axial plane. Furthermore, there are additional studies highlighting an increase in both surgery time (+10–15 min.) and costs in comparison to conventional instrumentation (USD300–650) [4,5]. This is in contrast to a questionably positive clinical effect. Until recently, there has been no clear evidence pointing to improved patients or improved functional outcomes in the literature, nor has there been a significantly reduced revision rate through the use of CAS [3,6].

Secondly, Patient Specific Instruments (PSI) were established in the mid-2000s. This technology is based on a preoperative imaging and computed planning of the component position. With the use of disposable patient individualized cutting jigs, the computer plan is transferred to the OR. Aside from improving the precision of the surgery (which is still under debate for PSI [7,8,9]), this technology was also designed to improve the intraoperative workflow, as it needs lesser instruments and creates a faster OR time [8,9,10]. Thienpont et al. summarized, in a systematic review and meta-analysis, that PSI improve the accuracy of femoral component alignment and global mechanical alignment, and they show a minimal benefit with regard to operative time and blood loss. However, this is, to their finding, at the cost of an increased risk of outliers for the tibial component alignment [8]. More to this, there are also critical reports pertaining to significant extra costs for imaging, planning, and the production of the PSI themselves [11,12]. Furthermore, again, a positive clinical effect on patients’ outcome could not yet be clearly demonstrated [13,14].

Most recently, in 2016, robotic technologies were introduced to TKA as the next evolutionary step of the conventional navigation. At present, active devices, such as a retracting handheld burr, or a saw assembled to a semi-active robotic-arm, were established. Therewith the alignment of the components and overall leg axis are not only passively monitored, but the technology assists the surgeon to precisely conduct the osteotomies and prevent over- or mal-resection of bone. This is intended to further improve precision and minimize errors. In the current literature, it is described that, compared with conventional manual-jig techniques, the robotic-assisted technique has been associated with increased accuracy and precision in coronal and sagittal alignment, better early functional outcomes, and reduced limb malalignment. [15,16]. Although, no difference in short-to-mid-term survivorship, aseptic loosening, periprosthetic joint infection, and complication rates were reported.

Additional to the potential for higher precision in osteotomies, with this technology it is now possible to virtually plan the implant position during surgery, based on knee models and soft tissue information, in order to individualize component position and reduce soft tissue releases. In a recent study, our group was able to show that, with an individualized restricted kinematic alignment protocol for robotic assisted TKA, soft tissue releases were only necessary in 10% of the cases, and only in severe valgus or posttraumatic cases [17]. However, the more features such a system contains, the more complex and time consuming it may become. Currently, there are only very limited studies available pertaining to the additional cost and effort resulting from such robotic devices [18]. The available literature concentrates on a comparison of a 90-day episode-of-care (EOC), observing conventional and robotic-assisted TKA, which provides the comparative costs alongside an overall benefit analysis for the technologies. However, the actual perioperative system-specific costs are not described in detail. More to this, different basic principles throughout the systems are to be distinguished: image-less vs. image-based technologies. In the first, the knee model is created during surgery, similar to the navigation; in the second, a preoperative imaging and segmentation is mandatory, and the surgery is pre-planned. These findings make it clear that different workflows, cost positions, and structures are to be expected. These aspects have not yet been investigated and described.

On the background of steadily increasing numbers of TKAs and healthcare costs, new technologies need to be carefully evaluated with regard to their cost-effectiveness and benefit for the patient. A new technology could lead to reduced costs (directly or due to lesser revision surgeries, for example), or it could improve the clinical outcome, and either result is worth the effort. For such analysis, however, the additional costs must be known and transparent.

Additionally, technologies have increasingly become marketing tools in the competitive health care market. The expectation of improved safety appears to attract new patients. Thus, many surgeons and health care administrations look closer into this field. In order to conduct a proper cost-benefit analysis, comparable numbers for each option are necessary.

At present, everything detailed in the available literature concerning the cost required, for these technologies, to implant TKA is limited, because it only evaluates one specific system in one specific setting. Most of the literature concerning navigation devices is rather old, and details surrounding these technologies may have changed over time. This makes it difficult and imprecise to compare the current available technologies with regard to their costs. Furthermore, potential differences in workflows between the technologies cannot be described, y as the reference technology may now differ. Additionally, data on robotic assisted surgery is still sparse, and often the study data include the learning curve, as the technology is relatively new.

The aim of the present study was to evaluate variables and differences in intraoperative workflows and related perioperative cost-profiles of four current assistive technologies, used to implant a TKA, in order to gain a comparison to conventional jig-based instrumentation in a standardized model.

## 2. Materials and Methods

### 2.1. Study Setting

Four expert centers were selected to study the record of the diverse workflows and expenditures related to five different methodologies used to implant a standard TKA:

1. Conventional jig-based TKA procedure: Centre Orthopedique Santy, Lyon, France and Annastift Hospital, Hannover Medical School, Hanover, Germany
2. Imageless navigated TKA (Nav.): articon Spezialpraxis für Gelenkchirurgie, Salem-Spital, Berne, Switzerland
3. Patient specific instrumentation for TKA (PSI): Clinic for Orthopaedics and Traumatology, Cantonal Hospital Winterthur, Winterthur, Switzerland
4. Imageless handheld robotic-assisted TKA (IL Robot): Annastift Hospital, Hannover Medical School, Hanover, Germany
5. Image-based robotic-arm-assisted TKA (IB Robot): Annastift Hospital, Hannover Medical School, Hanover, Germany.

Selection criteria for the expert centers were (1) more than 100 TKA implantations with the specific technology conducted per year by the participating surgeon in that institution and (2) instrument trays that were optimized with regard to the specific technology and surgeon’s workflow. All participating surgeons are specialized in knee replacement surgery. The surgeries included in the analysis were conducted in the summer of 2018.

### 2.2. Study Design

All cost calculations were based on the cost structure of the corresponding author’s institution located in Switzerland (in Swiss Francs), and these were converted to US-dollars at a rate of 1.05 (rounded to USD 10 each). As a baseline, the intraoperative workflow and necessary instruments for conventional TKA were evaluated as the average of nine observed procedures conducted by 3 different surgeons. For the comparative analysis, only parameters that differed between the methodologies were taken into account. Similar fixed costs for total knee arthroplasty were outlined, and it was agreed among the authors that they could be leveled because they were independent of the type of technology applied. It was the aim of this study to calculate the variable costs invoiced to the hospital, allowing our administration to calculate the return on investment of this technique and/or the additional costs. It was out of the scope of this study, because of its multicenter nature, to compare length of stay or costs of postoperative pain control, physiotherapy, or potential complications.

The analysis thus contained the following parameters with the rates listed:

1. Necessary additional preoperative imaging at USD 460 for a CT-scan
2. Preoperative planning time at USD 2.5/min (Orthopaedic surgeon salary)
3. Costs for surgical instruments at USD 160 per tray (processing, sterilization)
4. Cost for additional surgery time at USD 15/min
5. Costs for hardware leasing, technical support, and maintenance of equipment/case
6. Cost for additional disposals

The technology specific disposals were directly taken into account at the institution´s purchasing price at the Salem-Spital in Berne/Switzerland. The expenditures for the technical support of the devices were based on the current maintenance contracts of this hospital, and they were proportionally calculated based on an annual number of 125 TKAs. The acquisition of the hardware was not included into the evaluation. All are leasing models, and all costs are included into the service contracts (software updates, hardware leasing and maintenance, and support during surgery, including a mandatory on-site technician assistance for IB robot).

To analyze the different workflows of each methodology, and the surgery time, an independent observer visited the centers and prospectively reviewed several procedures. The surgeons were asked to select cases with primary osteoarthritis; ones that they would refer to be a standard TKA with minor need for soft tissue releases. For the conventional technique, serving as the baseline, three procedures, from three different surgeons, in two centers, were recorded, and the average time was measured both for each step and in total. With regard to the other technologies, only the differences that are technology specific were recorded, such as the time to position trackers for navigation, to register the bone anatomy and deformity into the navigation/robotic device, or the time for intraoperative computer assisted planning or navigation based validation of parameters, for example. Differences in the preparation time of the bone cuts, compared to the conventional jig-based average, were also recorded. In contrast, differences between the individual cases (need to resect osteophytes, soft tissue management) or surgeon specific workflows (exposure of the joint, closure etc.) were factored out. These steps of the procedure were referred to the average values of the conventional instrumentation. With regard to navigation and PSI, only one procedure classified as a standard TKA by the surgeon was reviewed. With regard to the robotic technologies, 10 consecutive surgeries were included into the analysis in order to rule out potential issues with the more recent technology and learning curve. All surgeons were well trained with their technology, and they were beyond the personal learning curve.

### 2.3. Statistical Analysis

For the intraoperative time per working step calculation, the average values were calculated from the different observed procedures (9 for conventional and 10 for each robotic group) using Windows Excel for Mac Version 15.34. Orthopedic salary per minute was calculated based on 250.000 CHF for 1800 work h/year.

## 3. Results

The average skin-to-skin surgery time for a conventional TKA was 59 min, with an additional 5 min of preoperative planning on standard radiographs. For conventional surgery, four instrument trays (1 basic instruments, 1 saw/drilling machine, 1 tibia instruments/trails, 1 femur instruments/trails) were used at a total cost rate of USD 640.

Compared to conventional, a standard TKA with usage of an image-less navigation system (PiGalileo, Smith & Nephew, Watford, UK) resulted in 14 min longer surgery time. This was due to additional navigation specific intraoperative steps, such as installation of trackers and reading in the bone anatomy, verification of bone cuts, alignment, and soft tissue tension as displayed in Table 1 (+USD 210).

**Figure 1:**
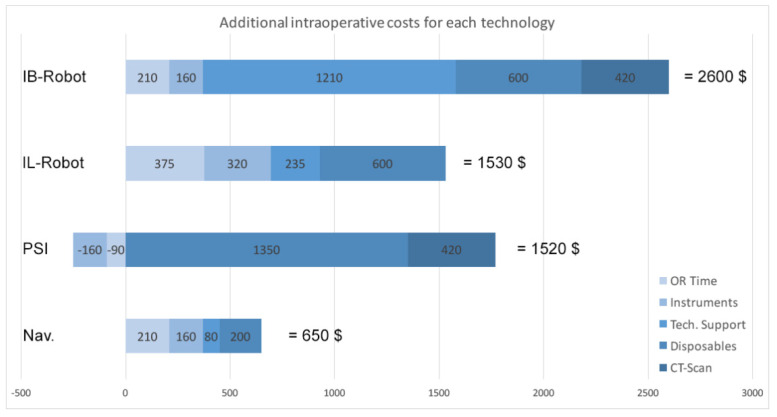
Graphic representation of the extra costs for each investigated technology used to implant a TKA, as compared to conventional standard procedure. The numbers indicate the different values in US-dollars per cost position.

Other surgery steps remained the same, such as intraoperative time to align the cutting guides and to conduct the osteotomies, for example. For the navigation, an extra instrument tray was necessary (+ USD 160). Technical support for the computer system was calculated at USD 80 additional cost per procedure. Navigation associated disposals added total additional costs of USD 200 per case (Figure 1).

The use of the PSI technology (MyKnee, Medacta International, Castel San Pietro, Switzerland) resulted in a 6 min reduction in surgery time, as the alignment of the cutting jigs takes less time when compared to conventional procedure (−USD 90) (Table 1). Also, the set of instruments could be reduced by one tray (−USD 160; tibia and femur instruments in one). In contrast, there were extra costs for a necessary preoperative CT scan (USD 420), and for the PSI blocks (USD 1350). Preoperative planning effort was slightly increased due to organization of the CT scan, the entry of the case into the proprietary PSI system, and the review of the planning proposal (+6 min, USD 15).

The imageless handheld robotic assisted technology (NAVIO, Smith & Nephew, Watford, UK) displayed the largest difference with regard to the intraoperative workflow, as compared to conventional standard. Despite installation of navigation trackers and registration of the bone anatomy, a surface model of the bone is also created during surgery, and all the planning of component sizes and position are made intraoperatively. This resulted in an average increase in surgery time by 25 min (Range 20–30 min) (USD 375) (Table 1). Two additional instrument trays were needed for the navigation and the handheld device (USD 320), with additional disposals worth USD 600, and technical support at USD 235 per surgery.

Image-based robotic arm-assisted TKA (MAKO, Stryker, Kalamazoo, MI, USA) showed an increase in surgery time, comparable to conventional navigation, of about 14 min on average (USD 210) (Table 1). Furthermore, two additional instrument trays were needed for the robotic device, whereas standard trays could be reduced by one as the implant sizes are known preoperatively and no conventional alignment instruments are necessary (USD 160). The major cost associated with the robotic arm technology was the technical support, including a mandatory on-site technician assistance for each procedure, resulting in a total of USD 1210 per surgery. In Addition, disposables expended USD 600 for each procedure. Lastly, a preoperative CT scan is also necessary for this technology (USD 420).

A comparative perioperative analysis of extra costs to conventional standard procedure is displayed in Figure 1.

## 4. Discussion

The focus of this study was to evaluate differences in intraoperative workflows and related cost-profiles of four current assistive technologies used to implant a TKA. To our knowledge, this is the only available analysis comparing competitive methods against the conventional instrumentation in order to transparently display additional costs and efforts.

Our major finding was as follows: all recently introduced technologies, used to assist and potentially improve surgical precision in TKA, increased the actual procedural costs and effort compared to standard conventional procedure. The biggest impact resulted from the technical support of computer-assisted technologies, and from the necessary additional disposables. Increased surgery time, and additional instrument trays, resulted in a minor effect on the overall costs. The image-based robotic-arm assisted procedure had the highest overall extra costs, followed with significant distance by the imageless handheld robotic system and the PSI technology, which were very close together in cost proximity. The conventional navigation had only a quarter of the additional costs for the robotic arm, but still expended USD 650 more than standard conventional TKA.

Regarding additional perioperative costs for robotic assisted technologies, there is currently only very sparse information in the literature, concentrating more on the overall cost-effectiveness, but not the actual additional costs. In a payer cost analysis for a 90 day episode-of-care (EOC), Cotter et al. described higher intraoperative costs for robotic assisted TKA (RA-TKA) compared to conventional techniques [18]. The “costs for the robot” were described to be around USD 800, but, interestingly, were not included in the cost calculation. This amount, of USD 800, differs significantly from our amount, of USD 2600, in total perioperative extra costs for IB robotic assistance. As Cotter does not give detailed information about their analytic methodology, no statement can be made about the different calculation bases. Thus, the actual cost positions, especially from the hospitals’ perspective, remain unclear in the current literature.

For imageless robotic-assistance, there is no data available on additional costs in the current literature. So, our calculation of USD 1530 represents a landmark pertaining to technology associated extra costs.

With regard to the classic computer navigation, the evidence based on costs and effort is better. Even though the literature is rather old, the findings are perfectly in line with the results of our study. Cerha et al. describe, in a Meta-analysis from 2009, additional costs for computer navigation at between EURO 300–395 (USD 360–479) in a 100 TKA per year calculation which includes maintenance of the system and additional OR time [19]. Koenig et al. calculated extra costs of about EURO 442 (USD 537) in the setting of a specialized arthroplasty unit [20]. These values are similar to the USD 650 we determined in our study. Nowak et al. even calculated additional USD 1500 in a literature review undertaken to create a cost effectiveness analysis [21]. Interestingly, there is no trend of falling costs detectable over time.

This same finding applies for the PSI technologies, as their described extra costs are consistent with our findings. Barrack et al. described cost savings, due to reduced OR time and instrument processing, to be around USD 322; however, they were overwhelmed by the USD 1500 additional cost of the MRI and the cutting guide [11]. This is comparable to the cost savings of around USD 250, and to the additional costs of USD 1770 in our study. Thienpont et al. determined the additional per case procedural costs to be around EURO 1142 (USD 1388) for the image based PSI [12].

The inclusion, in our calculation, of the costs for a preoperative imaging, which is necessary for PSI and image-based robotics, can be debated. This scan is usually performed preoperatively in an ambulatory setting and is thus not part of the direct hospital costs. The reimbursement is very much depending on the healthcare system; in many places the imaging is covered by health insurance with no extra costs for the hospital nor the patient.

Our second most important finding was as follows: all technologies were time consuming and increased OR time, except for the PSI. For the PSI, our finding is consistent with the current literature, however, the effect is only small [8,9]. In our study, the intraoperative benefit of the PSI in saving surgery time was more or less nullified by the increased preoperative organization and planning effort.

Also, the described elongated OR time for the conventional navigation is consistent with the literature. Cerha et al. reported, in his Meta-analysis, (exactly as we found in our study) 14 min. extra OR time [19]. Additionally, a very recent retrospective analysis of TKA procedures, conducted between 2016–2020, reported additional surgical time by 15 min. on average, compared to conventional TKA, for navigated TKA [22]. Therefore, although experience has increased over the last decade, not much has changed in these regards.

Regarding the intraoperative workflow and impact on OR time, again, the evidence on robotic-assisted technologies in TKA is limited. The aforementioned study conducted by Singh et al. reported an additional 11 min. for robotic assisted TKA over conventional [22]. This is in line with our finding of an extra 14 min.

Interestingly, in a direct comparison between the image-based and imageless robotics for unicompartimental-arthroplasty, the same effect that we observed is described: the imageless technology has a larger impact on OR time, with a wider scattering than the image-based system [23]. This is supported by other papers, specifying the additional OR time for IL Robot as 29 min [24], and, for the IB Robot, as only 5 min. extra [16].

It must be taken into account that only standard TKA cases with very minor balancing needs were included in our study. Potentially, in more complex cases, the technology based solutions, compared to conventional, might have a larger effect on the reduction of surgery time, as lesser adaptions are necessary after the cuts have been made. In our institution’s database, with more than 100 prospectively recorded robotic-assisted knee arthroplasties, we have observed a trend: surgery time is more constant and predictable when technology is used. The conventional technique, in contrast, can be faster, but the range in OR time is greater (unpublished data).

### 4.1. Limitations

Our chosen method, to not only evaluate additional cost positions, but also workflows and surgical steps, enables an easier adaption of our findings to different settings. However, the study has several limitations to be discussed. Firstly, it is a simplified analysis of some selected parameters. For example, differences in implant prices were not taken into account, and this is limiting because all evaluated technologies are implant specific and actual prices may differ. However, the implant prices vary too widely between countries, institutions, and suppliers, and, as a result, the transfer of the data would have been much more difficult. This same notion applies for the case complexity and the potential impact of different surgeons and institutions. By concentrating only on certain parameters, we tried to factor out these effects as much as possible in order to create a comparable standardized setting. Furthermore, the personnel costs were not taken into account. This could potentially influence the per minute rate in the OR, which can be easily adapted to specific settings.

Secondly, all cost calculations were based on a single institution’s cost structure pertaining to 125 TKAs a year. The acquisition of the hardware was not taken into account; we only counted the per surgery costs based on leasing models. We have observed that there are a wide range of finance models available, and these need to be accounted for when adapting the calculation model. The actual prices may differ significantly depending on regions and/or settings.

As a third limitation, one potentially relevant parameter for the cost analysis was not included: the set-up time for each technology. We were not able to measure the set-up times in a standardized methodology, and the institutional settings were too different, and, as a result, we could not compare them effectively. As these times only differ by a maximum of 5–15 min, the cost effect can be judged as minor.

Lastly, it must be stated: this study did not evaluate the cost effectiveness of these technologies; we only evaluated the perioperative extra costs and intraoperative effort. Thus, potential cost savings, with shortened hospital stay or faster rehabilitation, are not included in the data. Additionally, insurance status or reimbursement strategies, when using the technologies, were not further evaluated.

### 4.2. Practical Implications and Further Research

This study provides an overview of the differences between several assistive technologies used to implant a TKA in terms of intraoperative workflows and cost-profiles. This is valuable information for surgeons and hospital administrations in their efforts to calculate the real costs of these technologies and determine the subsequent return on investment of each technique. The outlined costs can easily be adapted to differing institutions and settings.

In addition to this, the specific costs for each technology serve as a base for further cost-effectiveness analyses. These can be used to calculate the economic value of technology in relation to the potential cost savings resulting from reduced revision rates, faster rehabilitation, and better quality of life for the patients.

## Figures and Tables

**Table 1:** Tabular representation of the differences in the intraoperative workflows for each investigated technology, as compared to conventional standard procedure.

	PSI	NAV	IL-Robot	IB-Robot
Install. Nav.	-	+3	+3	+3
Bone Reg.	-	+3	+7	+5
IntraOP Plan.	-	-	+7	+4
Fem. Prep.	−5	-	-	−2
Tib. Prep.	−1	-	-	−2
Gap Analysis	-	+2	+2	+2
Adaptions/Recuts	-	+2	+4	+2
Nav. Validation	-	+4	+2	+2
**Total mins.**	**−6**	**+14**	**+25**	**+14**

The numbers indicate the average additional or saved minutes per surgical step, respectively.
